# Case Report: Tislelizumab–induced panuveitis: a report of two cases

**DOI:** 10.3389/fphar.2025.1678663

**Published:** 2025-12-19

**Authors:** Huan Ding, Shuyuan Zhang, Lin Lin, Kaiyan Zhang

**Affiliations:** 1 Hainan Affiliated Hospital of Hainan Medical University (Hainan General Hospital), Haikou, China; 2 Department of Ophthalmology, Hainan General Hospital (Hainan Affiliated Hospital of Hainan Medical University), Haikou, China

**Keywords:** immune checkpoint inhibitors, tislelizumab, triamcinolone acetonide, uveitis, vogt-koyanagi-harada-like syndrome

## Abstract

**Background:**

Tislelizumab, an increasingly utilized immunotherapy for a range of malignancies, can induce a rare ocular immune-related adverse event resembling Vogt-Koyanagi-Harada-like syndrome (VKHLS). Management of complication requires careful balancing with ongoing systemic anti-tumor therapy.

**Case Presentation:**

This report describes two cancer patients who developed severe bilateral vision loss following Tislelizumab treatment. Multimodal ocular imaging confirmed the diagnosis of immune checkpoint inhibitors (ICIs)-associated uveitis, specifically a VKHLS. Both patients were managed with discontinuation of Tislelizumab and local corticosteroid therapy-transcutaneous periocular injection of triamcinolone acetonide (TA) and topical steroid eye drops, without systemic corticosteroids. Their uveitis resolved rapidly, with recovery of visual acuity.

**Conclusion:**

Prompt Tislelizumab discontinuation combined with local corticosteroid therapy-transcutaneous periocular TA injections, and topical steroid eye drops may constitute an effective management strategy for ICIs-induced uveitis. This approach is particularly valuable for cancer patients in whom systemic corticosteroids are contraindicated or unsuitable.

## 1 Introduction

Immune checkpoint inhibitors (ICIs) represent a transformative class of agents in cancer immunotherapy (Leach et al., 1996). Under physiological conditions, immune checkpoint molecules prevent excessive T-cell activation. Tumor cells, however, can exploit this mechanism by upregulating checkpoint molecules and/or fostering an immunosuppressive microenvironment to evade immune surveillance. ICIs counteract this evasion by blocking checkpoint molecules such as PD-1, PD-L1, and CTLA-4, thereby releasing inhibitory signals on T cells and reactivating intrinsic antitumor immunity paradigm shift in oncology (Dine et al., 2017).

Despite their efficacy, the potentiation of antitumor immunity by ICIs can trigger off-target autoimmune reactions against healthy tissues, termed immune-related adverse events (irAEs) (Baxi et al., 2018). Among these, ocular irAEs (OirAEs) remain under-recognized, affecting approximately 2.8%–4.3% of ICIs recipients (Wu et al., 2024). Clinical manifestations include uveitis, dry eye, optic neuritis, and conjunctivitis (Kim et al., 2023; Wu et al., 2024). Uveitis accounts for 15.1% of OirAEs, with anterior uveitis (43%) predominating, followed by panuveitis (37%) and posterior uveitis (19%) (Sun Mm Md et al., 2020; Bomze et al., 2022).

Current understanding of OirAEs derives largely from case reports and small case series. Their low incidence and heterogeneous clinical presentations contribute to under-recognition by both clinicians and patients, often leading to delays in diagnosis and treatment. Such delays can significantly increase the risk of irreversible vision loss.

## 2 Case description

### 2.1 Case 1

**FIGURE 1:**
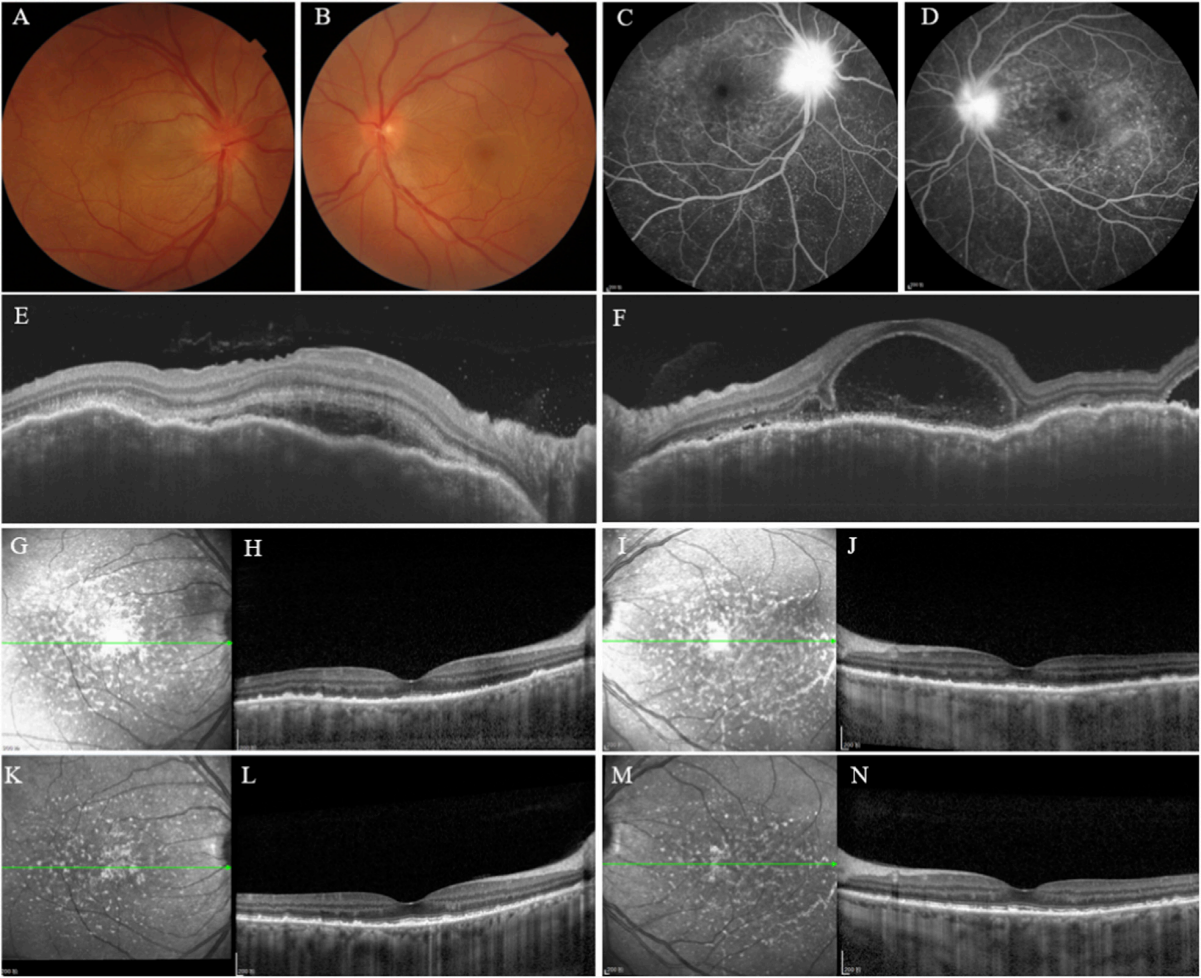
(Case1) **(A,B)** Fundus photographs of OD and OS: showing bilateral optic disc edema with indistinct margins and posterior pole retinal edema. **(C,D)** FFA of OD and OS: demonstrating multiple punctate hyperfluorescent foci with pooling in the posterior pole, late-phase multifocal lacunar dye leakage, hyperfluorescent staining of the optic discs with blurred borders. **(E,F)** OCT of OD and OS: showing bilateral choroidal folds and serous retinal detachment. **(G–J)** 3-week post-treatment: infrared reflectance of OCT showing punctate hyperreflective lesions in the posterior pole of OD **(G)** and OS **(I)**; OCT demonstrating SRF resolution and deposits within the RPE layer of OD **(H)** and OS **(J)**. **(K–N)** 3-month post-treatment: infrared reflectance of OCT showing a reduction in hyperreflective lesions of OD **(K)** and OS **(M)**; OCT demonstrating decreased punctate hyperreflective deposits within the RPE layer of OD **(L)** and OS **(N)**.

A 55-year-old female presented to our hospital’s gynecology department in April 2022 with microsatellite instability-high (MSI-H) metastatic endometrial adenocarcinoma. She underwent surgical resection followed by adjuvant chemotherapy with the paclitaxel-carboplatin (TC) regimen combined with the ICIs Tislelizumab (200 mg). From May 2022 to April 2024, she completed 5 cycles of TC plus Tislelizumab, followed by 19 cycles of Tislelizumab maintenance therapy. One week after the 22nd dose (February 2024), she experienced a decline in bilateral visual acuity and presented to ophthalmology 1 month after symptom onset. Ophthalmic Examination Findings (Table 1): Best-corrected visual acuity (BCVA) was 0.01 logMAR (Snellen equivalent of 20/25) in the right eye (OD) and 0.70 logMAR (Snellen equivalent of 20/100) in the left eye (OS). Normal IOP OU by non-contact tonometry. Pupils measured 3*3 mm in diameter, with a relative afferent pupillary defect (RAPD) in the OS. Slit-lamp examination showed bilateral mixed conjunctival hyperemia, moderate dust-like keratic precipitates (KP), and anterior chamber cells (+) (Binder et al., 2025). Bilateral lens opacities were graded as C1N3P1 (Chylack et al., 1993), with dust-like pigment deposition on the anterior capsule surface. Fundus photography revealed optic disc edema with blurred margins and posterior pole retinal edema in both eyes (Figures 1A,B). Fundus fluorescein angiography (FFA) showed multiple punctate hyperfluorescent foci with pooling in the posterior pole of OU, late-phase multifocal lacunar dye leakage, and hyperfluorescent staining of the optic discs with blurred borders (Figures 1C,D). Optical coherence tomography (OCT) showed choroidal folds and subretinal fluid (SRF) OU (Figures 1E,F). A diagnosis of ICIs-associated uveitis, manifesting as Vogt-Koyanagi-Harada-like syndrome (VKHLS). Following multidisciplinary consultation with the Gynecology Oncology department, discontinuation of Tislelizumab immunotherapy was recommended. Ophthalmic treatment comprised transcutaneous periocular triamcinolone acetonide (TA) injection (Zhang et al., 2018), in combination with topical corticosteroids and mydriatic drops OU. The table below summarizes the patient’s ophthalmic examination findings and grading at baseline, 3 weeks, and 3 months follow-up, based on the Common Terminology Criteria for Adverse Events (CTCAE) version 5.0. At the 3-week visit, BCVA was 0.10 logMAR (Snellen equivalent of 20/25) OU, IOP was within normal range OU. Slit-lamp examination showed improvement of mixed conjunctival hyperemia, mild dust-like KP, and persistence of anterior chamber cells (+). Lens opacities remained graded as C1N3P1 with dust-like pigment on the anterior capsule. Fundus examination revealed disc and retinal edema. OCT showed punctate hyperreflective lesions in the posterior pole of OU (Figures 1G,I) and demonstrated the resolution of SRF with deposits within the retinal pigment epithelium (RPE) layer of OU (Figures 1H,J). Then a second transcutaneous periocular TA injection (40 mg) was administered, along with continued topical corticosteroid and cycloplegic eye drops. At the 3-month follow-up, BCVA was improved to 0.00 logMAR (Snellen equivalent of 20/20) OU, IOP was within normal range OU. Slit-lamp examination showed resolution of mixed conjunctival hyperemia, mild dust-like KP, anterior chamber cells (−). Lens opacities were graded as C1N3P1 without pigment deposition on the anterior capsule. OCT showed a reduction in hyperreflective lesions of OU (Figure 1K,M) and demonstrated decreased punctate hyperreflective deposits within the RPE layer of OU (Figure 1L,N).

**TABLE 1:** Case 1 eye signs and grading.

Metric	Baseline		3 Weeks		3 Months	
	OD	OS	OD	OS	OD	OS
BCVA (Snellen/logMAR)	20/25 0.10	20/100 0.70	20/25 0.10	20/25 0.10	20/200 0.00	20/200 0.00
IOP (mmgh)	12	9	14	12	15	13
AC cell	+	+	+	+	-	-
Lens (LOCS III)	C1N3P1	C1N3P1	C1N3P1	C1N3P1	C1N3P1	C1N3P1
Vitreous haze	Present	Present	Improving	Improving	Resolved	Resolved
Optic disc status	Disc with blurred margins and retinal edema	Disc with blurred margins and retinal edema	Disc with blurred margins and retinal edema	Disc with blurred margins and retinal edema	Complete absorption	Complete absorption
OCT metrics	Choroidal folds and SRF	Choroidal folds and SRF	Subretinal fluid absorption with punctate hyperreflective deposits at RPE level	Subretinal fluid absorption with punctate hyperreflective deposits at RPE level	Complete fluid absorption, reduced deposits	Complete fluid absorption, reduced deposits
CTACE version 5.0		Grade 2: Anterior uveitis with 1 + or 2 + cells				

SRF: subretinal fluid. CTACE: common terminology criteria for adverse events.

### 2.2 Case 2

**FIGURE 2:**
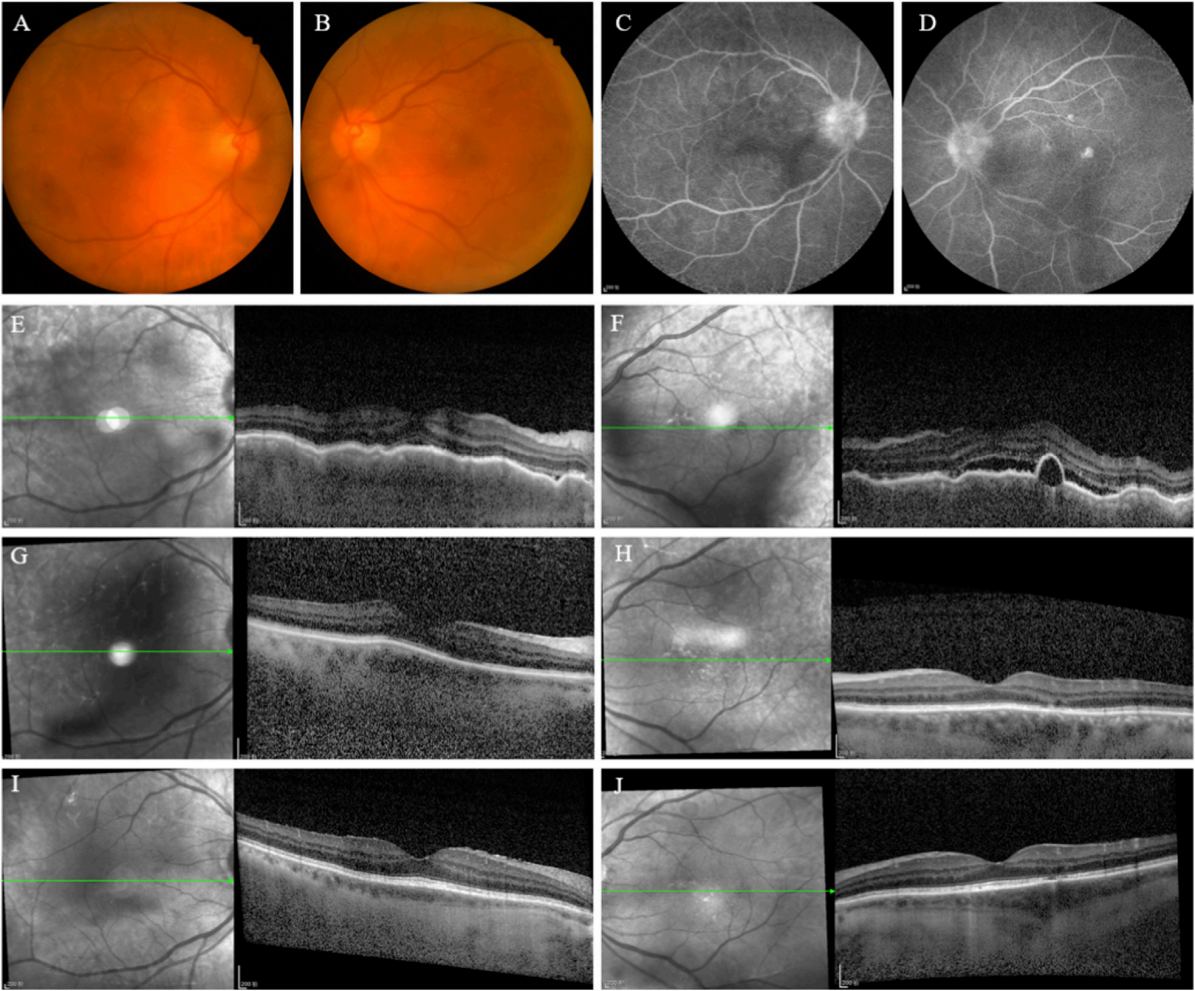
(Case2) **(A,B)** Fundus photographs of OD and OS: showing bilateral optic discs with a reddish hue and unclear boundaries, and retinal edema in the posterior pole. **(C,D)** FFA images of OD and OS: demonstrating bilateral optic disc hyperfluorescence with blurred margins. In the early phase, multifocal punctate hyperfluorescent foci were observed in the posterior pole, which progressed to fluorescein leakage and pooling in the late phase. **(E,F)** OCT of OD and OS: showing bilateral choroidal folds and serous retinal detachment. **(G,H)** 3-week post-treatment: showing bilateral choroidal folds and resolution of SRF. **(I,J)** 3-month post-treatment: demonstrating mild irregularity of the ellipsoid zone (EZ) in OD and near-normal retinal morphology in OS.

A 66-year-old male was diagnosed with extensive-stage small cell lung cancer (ES-SCLC), stage IV (cT4N2M1), with pleural metastasis in June 2024. He initiated first-line chemotherapy with etoposide-carboplatin (EC) combined with Tislelizumab (200 mg) immunotherapy. Following his fifth chemotherapy-immunotherapy cycle in September 2024, the patient developed bilateral visual decline and was referred for ophthalmic evaluation. Ophthalmic Examination Findings (Table 2): BCVA was 1.00 logMAR (Snellen equivalent of 20/200) OU, IOP was within normal range OU. Pupils measured 3*3 mm in diameter, with sluggish direct and indirect light reflexes OU, and no RAPD was detected. Slit-lamp examination showed bilateral mixed conjunctival hyperemia, numerous pigmented KPs, anterior chamber cells (4+) (Binder et al., 2025). Bilateral lens opacities were graded as C2N3P0 (Chylack et al., 1993), with dust-like pigment deposits on the anterior capsules. Fundus photography revealed hyperemic optic discs with blurred margins and posterior pole retinal edema (Figures 2A,B). FFA showed bilateral optic disc hyperfluorescence with blurred margins, early-phase imaging displayed multiple punctate hyperfluorescent foci in the posterior pole, which progressed to leakage and pooling in the late phase (Figures 2C,D). OCT demonstrated choroidal folds and SRF OU (Figures 2E,F). Thus, he was diagnosed with ICIs-associated uveitis, as VKHLS. Following multidisciplinary consultation with the Respiratory Oncology Tislelizumab was discontinued. Ophthalmic treatment included a transcutaneous periocular TA injection (40 mg), combined with topical corticosteroid and mydriatic eye drops. The table below summarizes the patient’s ophthalmic examination findings and grading at baseline, 3 weeks, and 3 months follow-up, based on the CTCAE version 5.0. At the 3-week follow-up, BCVA improved to 0.20 logMAR (Snellen equivalent of 20/32) OU, IOP was within normal range OU. Slit-lamp examination showed improvement of mixed conjunctival hyperemia, moderate pigmented KP, and anterior chamber cells (2+). Lens opacities remained graded as C2N3P0 with dust-like pigment on the anterior capsules. Fundus examination indicated resolution of optic disc and retinal edema. OCT revealed marked improvement in choroidal folds and complete absorption of SRF (Figures 2G,H). The patient received a second transcutaneous periocular TA injection (40 mg) with continued topical corticosteroid and mydriatic eye drops. At 3-month follow-up, BCVA further improved to 0.10 logMAR (Snellen equivalent of 20/25) OU, IOP was within normal range OU. Anterior chamber cells resolved completely. Lens opacities were graded as C2N3P0 without pigment deposition on the anterior capsule surface. OCT showed mild irregularity of the ellipsoid zone OD (Figures 2I,J).

**TABLE 2:** Case 2 eye signs and grading.

Metric	Baseline		3 Weeks		3 Months	
	OD	OS	OD	OS	OD	OS
BCVA (Snellen/logMAR)	20/200 1.00	20/200 1.00	20/32 0.20	20/32 0.20	20/25 0.10	20/25 0.10
IOP (mmgh)	8	9	11	13	12	10
AC cell	4+	4+	2+	2+	-	-
Lens (LOCS III)	C2N3P0	C2N3P0	C2N3P0	C2N3P0	C2N3P0	C2N3P0
Vitreous haze	Present	Present	Improving	Improving	Resolved	Resolved
Optic disc status	Disc with blurred margins and retinal edema	Disc with blurred margins and retinal edema	Resolution of optic disc and retinal edema	Resolution of optic disc and retinal edema	Complete absorption	Complete absorption
OCT metrics	Choroidal folds and SRD	Choroidal folds and SRD	Improvement in choroidal folds and complete absorption of SRF	Improvement in choroidal folds and complete absorption of SRF	Mild irregularity of the ellipsoid zone	Complete fluid absorption, reduced deposits
CTACE version 5.0		Grade 4: Best corrected visual acuity of 20/200 or worse in the affected eye				

## 3 Discussion

Tislelizumab is a humanized monoclonal antibody targeting PD-1, developed independently in China, and classified as an ICIs. Tislelizumab demonstrated a clinically meaningful improvement in objective response rate (ORR) in patients with previously treated, locally advanced unresectable or MSI-H tumors, with a favorable safety profile (Li et al., 2024). Furthermore, clinical trials established its efficacy in significantly prolonging overall survival and progression-free survival in patients with ES-SCLC when combined with chemotherapy, while maintaining manageable safety (Cheng et al., 2024).

We report two cases of immune-related Vogt-Koyanagi-Harada-like syndrome (irVKHLS) following combination therapy with Tislelizumab and platinum-based chemotherapy. Case 1 involved a patient with MSI-high endometrial adenocarcinoma treated with TC, while Case 2 had ES-SCLC treated with EC. Both patients received Tislelizumab at 200 mg. This specific form of immune-related panuveitis is characterized by diffuse choroidal thickening and serous retinal detachments (Chang et al., 2025). Panuveitis developed at 66 weeks after treatment initiation in Case 1 and at 15 weeks in Case 2. Although most OirAEs occur within 24 weeks, delayed-onset uveitis has been reported as late as 72 weeks, and even up to 2 years after starting treatment (Shahzad et al., 2021; Martens et al., 2023; Wang et al., 2024), which is consistent with the presentation in Case 1. Given the absence of documented uveitis induced by paclitaxel, carboplatin, or etoposide monotherapy in the literature, we applied the Naranjo Algorithm for causality assessment of adverse drug reactions. This algorithm classifies causality as follows: a score >9 indicates a “definite” relationship, 5–8 indicates “probable”, 1–4 indicates “possible”, and ≤0 indicates “doubtful”. Both cases scored 7, corresponding to a “probable” rating (More et al., 2024). Similarly, the WHO-UMC system—which categorizes causality as “Certain”, “Probable”, “Possible”, “Unlikely”, “Conditional”, or “Unassessable”—also assigned a “probable” causality assessment for both cases (Sakiris et al., 2021). A review of concomitant medications further supported Tislelizumab as the causative agent.

For patients with malignancies, cancer-associated retinopathy (CAR) and bilateral diffuse uveal melanocytic proliferation (BDUMP) represent important differential diagnoses. However, key features in our cases argued against these conditions. CAR typically presents with retinal thinning (Brossard-Barbosa et al., 2023) and is unresponsive to corticosteroids (Brossard-Barbosa et al., 2023), unlike the pronounced edema and significant improvement following periocular steroid injection observed in these two cases. Moreover, VKH-like manifestations are most frequently observed in melanoma patients treated with ICIs not with CAR (Martens et al., 2023). Furthermore, while both patients had bilateral vision loss and subretinal fluid, the absence of rapid cataract progression (Drača et al., 2023), characteristic fundus changes, and pathognomonic imaging findings—such as outer retinal disruption on OCT or a “giraffe” pattern on near-infrared imaging (Sarigul Sezenoz et al., 2025)—effectively ruled out BDUMP. In addition, although the pathophysiology of ICIs-associated uveitis involves systemic autoimmunity against melanocytes, and VKH-like panuveitis is frequently documented in Melanoma-associated retinopathy (MAR) (Crosson et al., 2015; Bricout et al., 2017; Obata et al., 2019; Enomoto et al., 2021; Minami et al., 2021; Monferrer-Adsuara et al., 2021), comprehensive systemic evaluations revealed no evidence of melanoma in our two cases. Study limitations include the unavailability of ERG and anti-retinal antibody testing. Nonetheless, the collective clinical and imaging findings, combined with the treatment response, confidently exclude CAR, BDUMP, and MAR.

The U.S. National Cancer Institute’s Common Terminology Criteria for Adverse Events (CTCAE) version 5.0 provides a standardized grading system (Grades 1-5) for immune-related toxicities, guiding the diagnosis and management of irAEs, including OirAEs^1^ (Institute, 2017). Accordingly, professional societies such as the American Society of Clinical Oncology (ASCO), the National Comprehensive Cancer Network (NCCN), and the Society for Immunotherapy of Cancer (SITC) have established uveitis management guidelines based on CTCAE grading (Thompson et al., 2019; Brahmer et al., 2021; Schneider et al., 2021). The management of irAEs is primarily determined by the CTCAE severity grade and the specific organ system affected (Schneider et al., 2021; Olsen et al., 2022). These consensus guidelines recommend systemic glucocorticosteroids (GCs) for irAEs of Grade 2 or higher severity. Consequently, systemic GC therapy was the guideline-recommended treatment for both patients described here, who presented with Grade 2 and Grade 4 OirAEs, respectively. However, the use of GCs requires careful risk-benefit analysis, as they can antagonize the anti-tumor efficacy of ICIs through several mechanisms, including suppression of antigen presentation, inhibition of lymphocyte trafficking, and impairment of immune-mediated tumor cell killing (Jove et al., 2019; Goodman et al., 2023). In these specific cases, due to the patients’ significant concerns regarding potential systemic steroid-related complications, transcutaneous periocular TA injections were selected as the preferred treatment following a thorough discussion. This peripheral route of administration (transcutaneous/transconjunctival) delivers the long-acting glucocorticoid locally, offering targeted efficacy and a favorable safety profile compared to intravitreal delivery (Nozik, 1972). Subsequent follow-up examinations at 1 year for Case 1 and 6 months for Case 2 confirmed stable ocular findings in both patients.

Current consensus guidelines recommend discontinuing ICIs in patients who develop Grade 2 or higher OirAEs (Thompson et al., 2019; Brahmer et al., 2021; Schneider et al., 2021). Consistent with this approach, ICI therapy was withdrawn in both of the present cases, which involved Grade 2 and Grade 4 OirAEs, respectively. Reassuringly, neither patient showed tumor progression following ICI cessation. It should be noted, however, that while discontinuing ICIs may help alleviate ocular symptoms, the extended half-life of these agents limits the utility of withdrawal as a standalone strategy for controlling persistent immune activation. Notably, recent expert consensus has advocated for a revised approach to irVKHLS, emphasizing the use of topical, periocular, and/or intraocular corticosteroids without the mandatory discontinuation of ICIs (Chang et al., 2025). This updated treatment paradigm provides a more optimized framework for the clinical management of ICI-induced panuveitis.

## 4 Conclusion

Clinicians should maintain a high index of suspicion for OirAEs in patients receiving PD-1 inhibitors. When visual symptoms occur, prompt referral to ophthalmology and initiation of a multidisciplinary management strategy are essential. Rather than systemic GCs, this approach not only averted systemic side effects but also mitigated potential interference with the tumor process. While further clinical evidence is nonetheless required to confirm these observations.

## Data Availability

The original contributions presented in the study are included in the article/supplementary material, further inquiries can be directed to the corresponding author.

## Footnotes

1
https://dctd.cancer.gov/research/ctep-trials/for-sites/adverse-events/ctcae-v5-5x7.pdf
